# Supplementary figures and images for: Gold Nanoparticles Enhance the Tumor Growth-Suppressing Effects of Cetuximab and Radiotherapy in Head and Neck Cancer In Vitro and In Vivo
Source: Cancers (Basel). 2023 Dec 3;15(23):5697. doi: 10.3390/cancers15235697 (PMC10705767; doi:10.3390/cancers15235697)

## Supplementary Figure S1

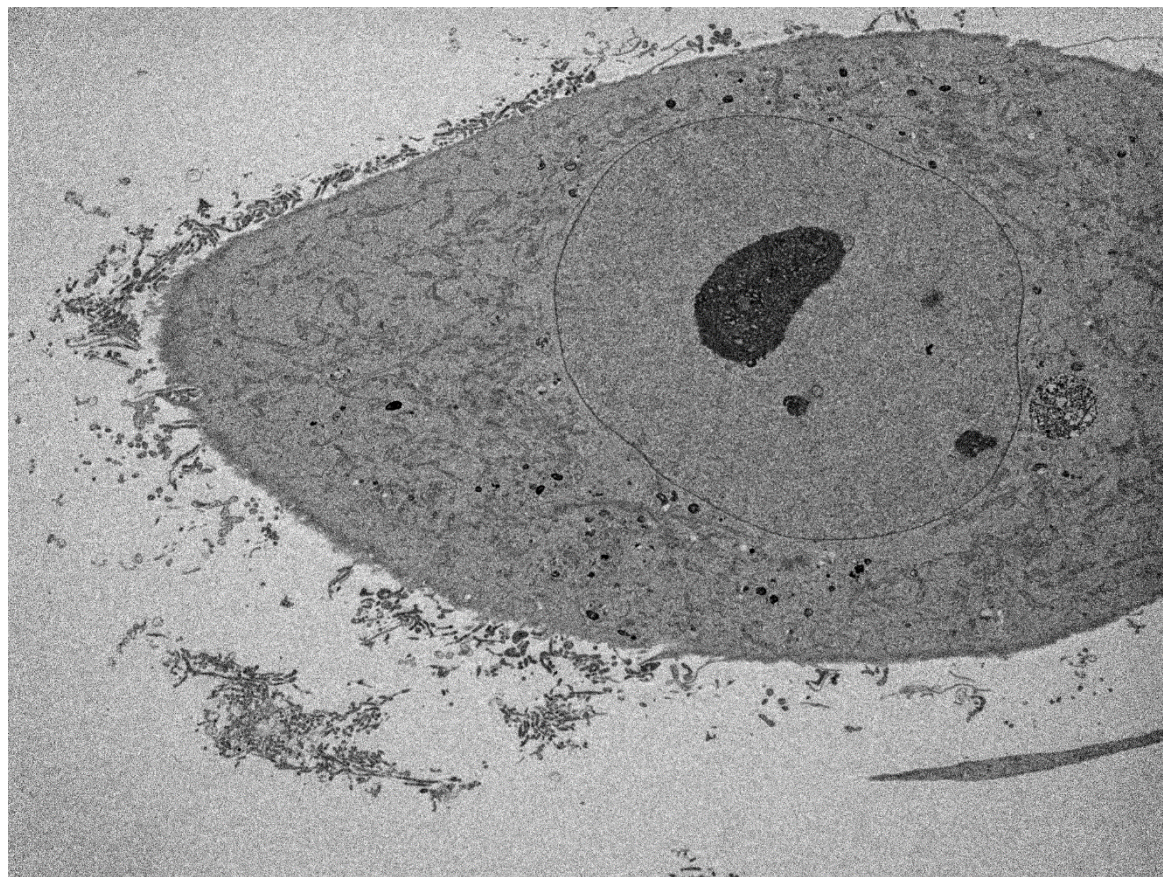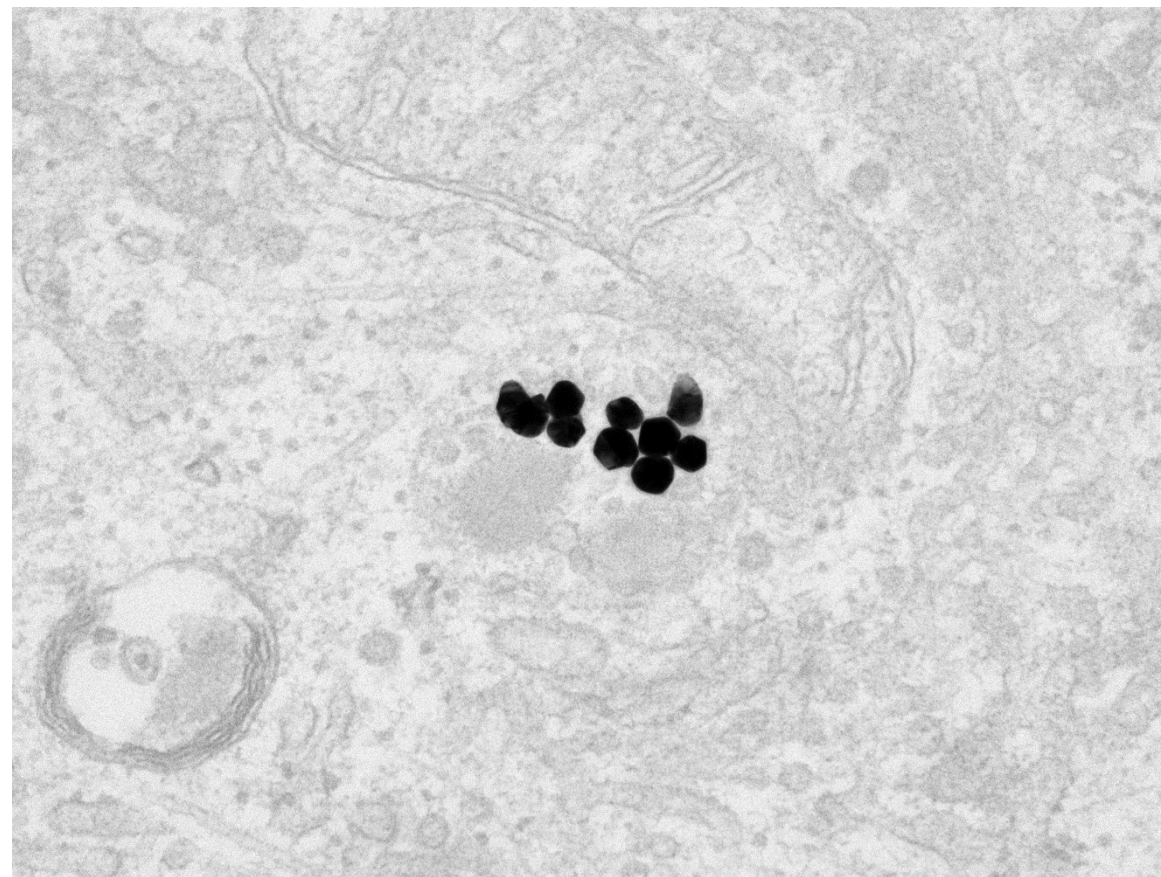

Representative TEM images. AuNP clusters consisted of several AuNPs.

Supplement: Supplementary file 1 [file cancers-15-05697-s001.zip › cancers-2723747-supplementary.pdf]
